# Correction: Mitochondrial defects caused by PARL deficiency lead to arrested spermatogenesis and ferroptosis

**DOI:** 10.7554/eLife.103278

**Published:** 2024-09-13

**Authors:** Enrico Radaelli, Charles-Antoine Assenmacher, Jillian Verrelle, Esha Banerjee, Florence Manero, Salim Khiati, Anais Girona, Guillermo Lopez-Lluch, Placido Navas, Marco Spinazzi

**Keywords:** Mouse

Radaelli E, Assenmacher C-A, Verrelle J, Banerjee E, Manero F, Khiati S, Girona A, Lopez-Lluch G, Navas P, Spinazzi M. 2023. Mitochondrial defects caused by PARL deficiency lead to arrested spermatogenesis and ferroptosis. *eLife* **12**:e84710. doi: 10.7554/eLife.84710.

Published 28 July 2023

We were notified by eLife journal and via PubPeer of three errors.

The first one is in Figure 4A, where the ACTB band is incorrect. The one depicted is actually the ACTB band of Figure 6B. However, the correct actin band has been provided as part of the original source data files. This unintentional duplication occurred during panel assembly due to the very high similarity of these western blots which makes them not easy to distinguish. We corrected Figure 4 accordingly. The actin band in the corrected Figure 4A now matches the original images in the published Figure 4—source data 1. This mistake does not affect to any extent the results or interpretation of this experiment and the conclusions of this work.

The corrected Figure 4 is shown here:

**Figure 1:**
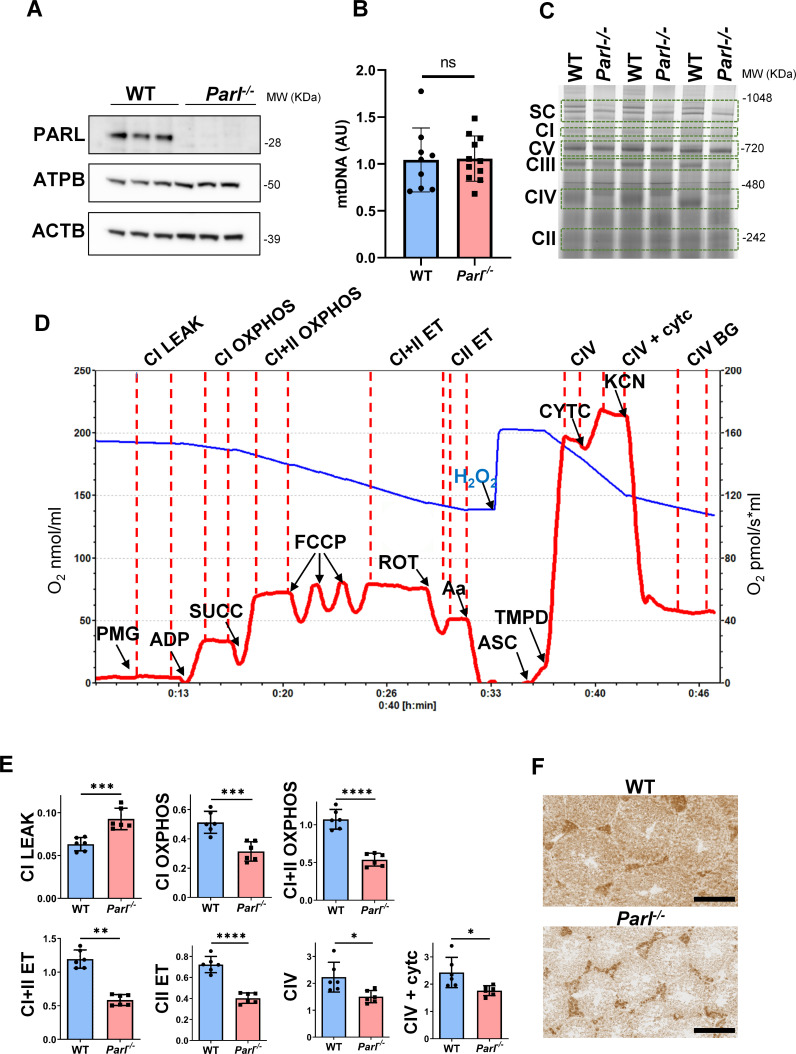


The originally published Figure 4 is shown for reference:

**Figure 2:**
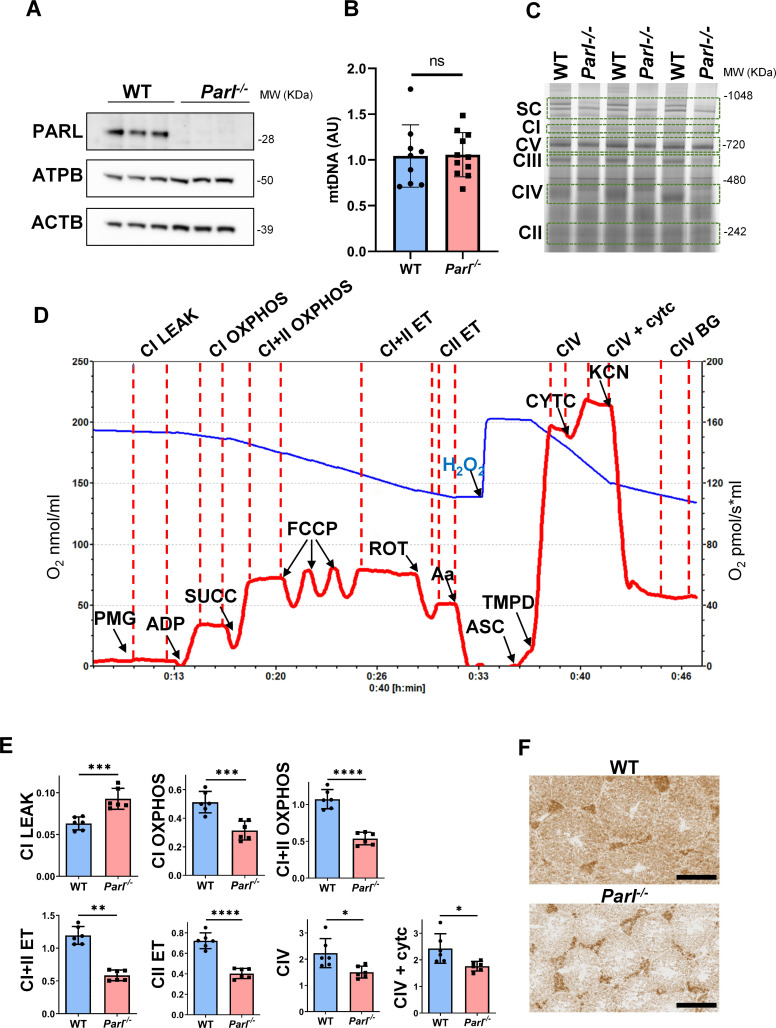


The second mistake is in the legend of Figure 4A. The age of mice for this experiment was 5 weeks, not 6. We performed the same analysis at later time points, obtaining identical results.

Corrected sentence in Figure 4A legend:

(A) Immunoblots of testis lysates from 5-week-old WT and *Parl^−/−^* mice

Original published sentence in Figure 4A legend:

(A) Immunoblots of testis lysates from 6-week-old WT and *Parl^−/−^* mice

The third error is the duplication of panels WT and *Ttc19^-/-^* in Figure 3—figure supplement 1. This mistake stems from the fact that sections of WT and *Ttc19^-/-^* testis were placed on the same slide, and images of WT and *Ttc19^-/-^* have both been taken by mistake from the *Ttc19^-/-^* section. This unintentional duplication occurred because of indistinguishable AIF-1 staining in the two genotypes. This error has been corrected by replacing the originally published WT image with an image captured from the actual WT section. The WT and Ttc19-/- mouse testes are both histologically normal and display comparable AIF-1 IHC results. Therefore, this mistake does not affect the results or interpretation of this experiment and the conclusions of this work.

The corrected version of Figure 3—figure supplement 1 is shown here:

**Figure 3:**
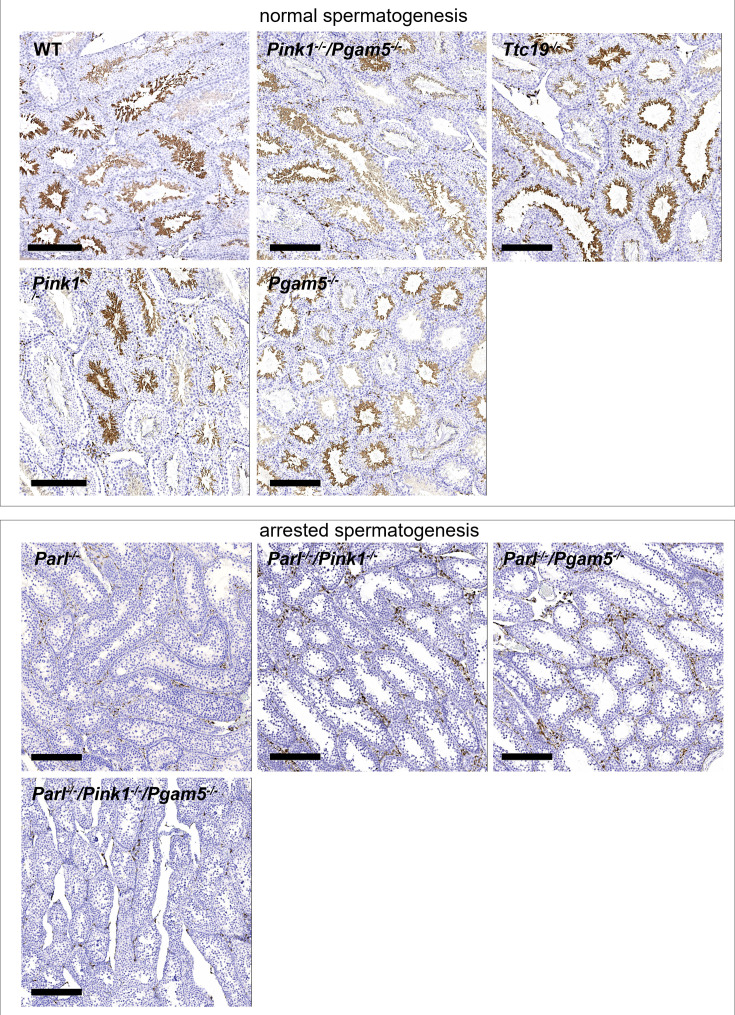


The originally published Figure 3—figure supplement 1 is shown for reference:

**Figure 4:**
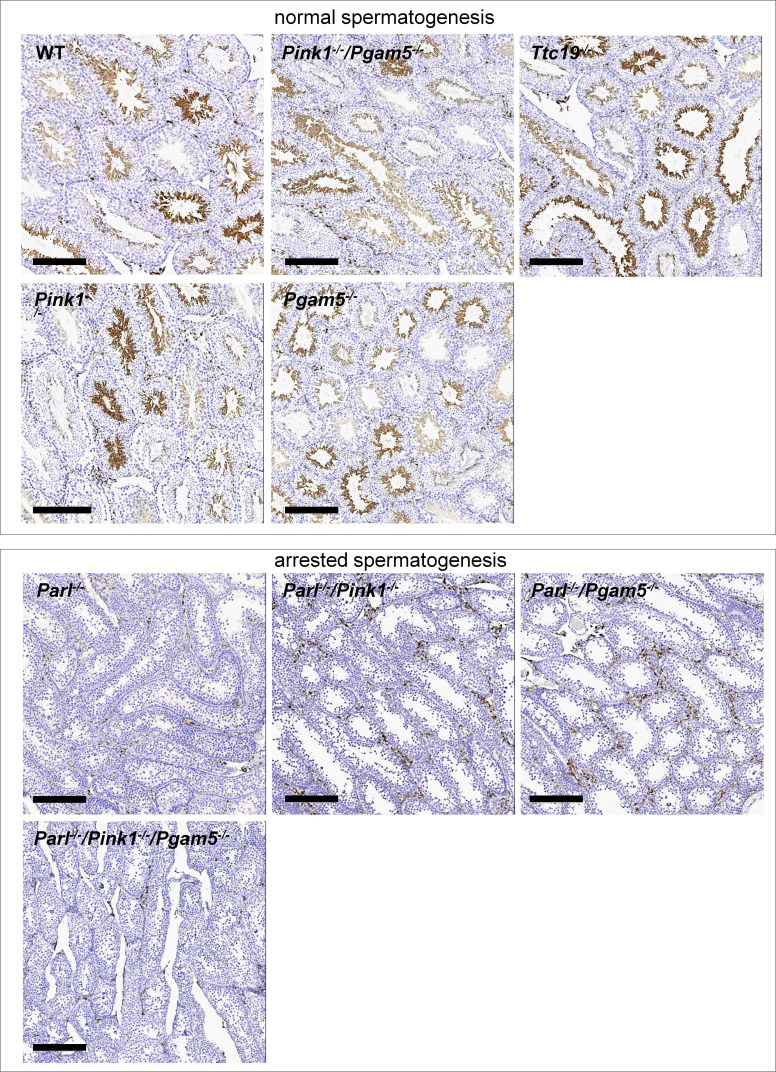


We thank the users of PubPeer for alerting us of this issue. The article has been corrected accordingly.

