# Supplementary figures and images for: Mitochondrial defects caused by PARL deficiency lead to arrested spermatogenesis and ferroptosis
Source: eLife. 2023 Jul 28;12:e84710. doi: 10.7554/eLife.84710 (PMC10519710; doi:10.7554/eLife.84710)

**Figure 3A-source data**

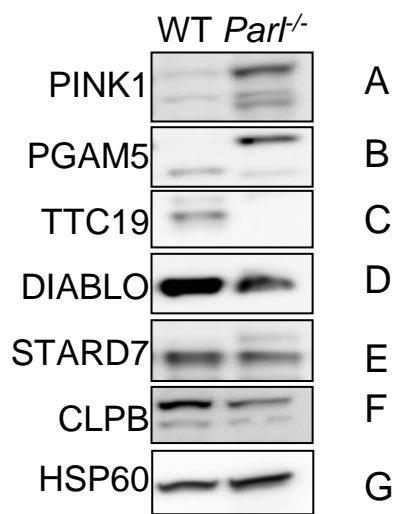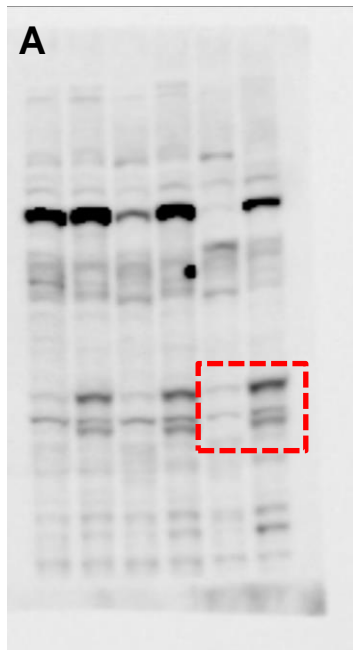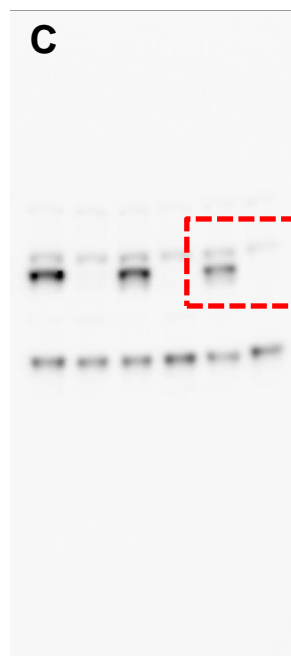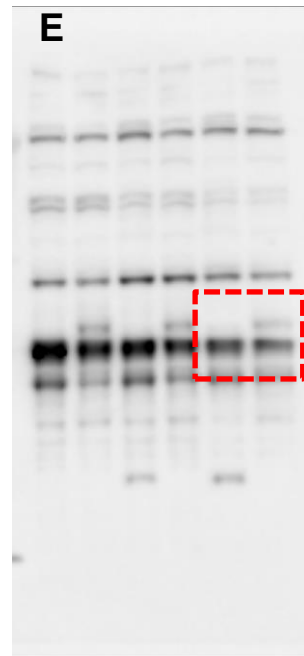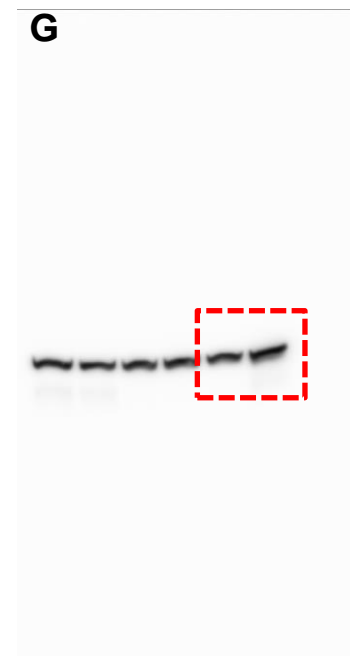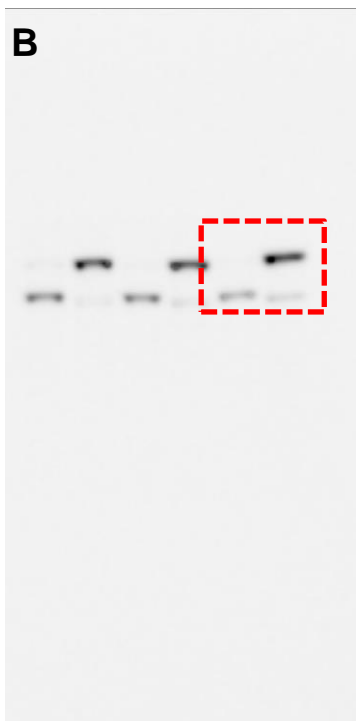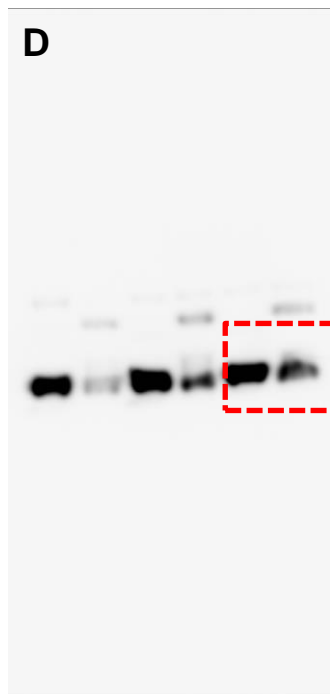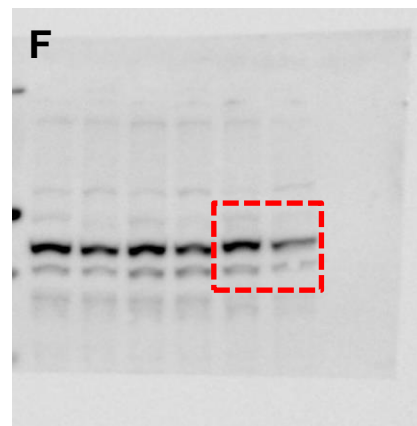

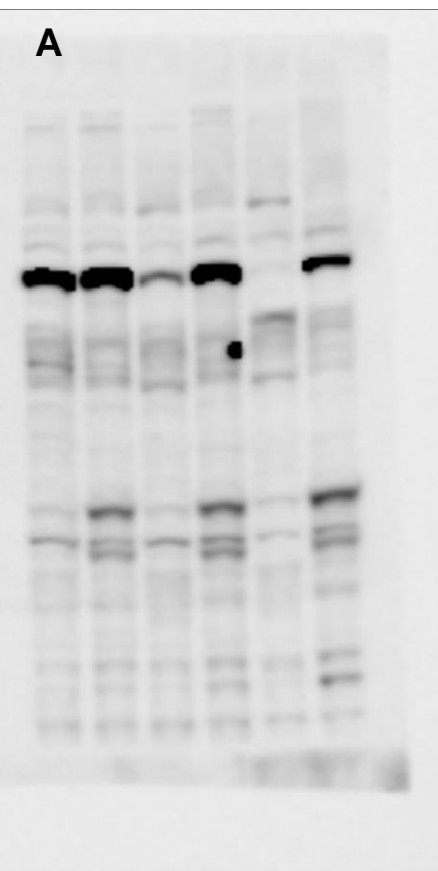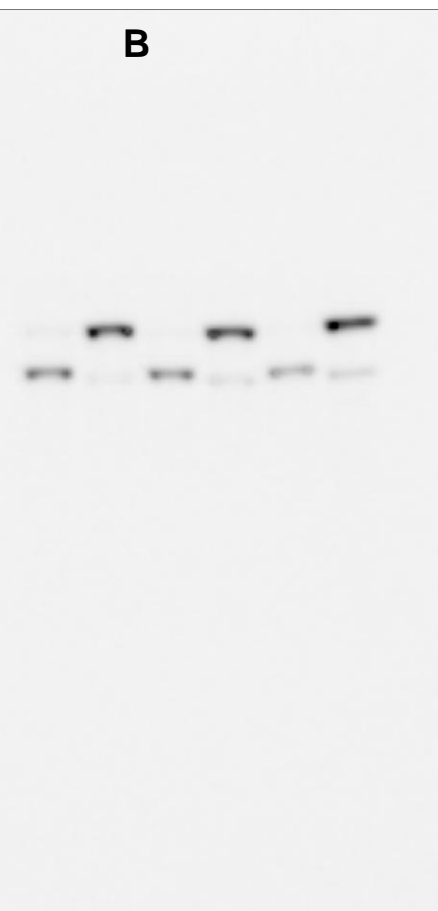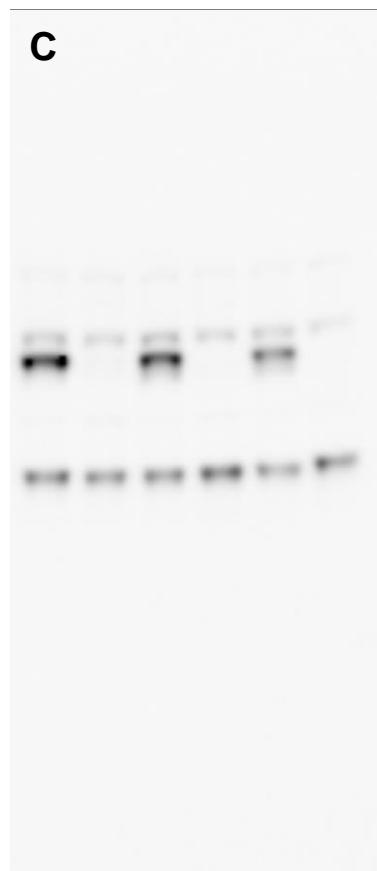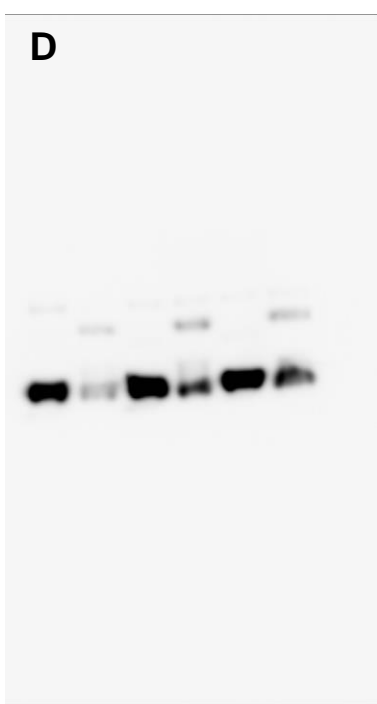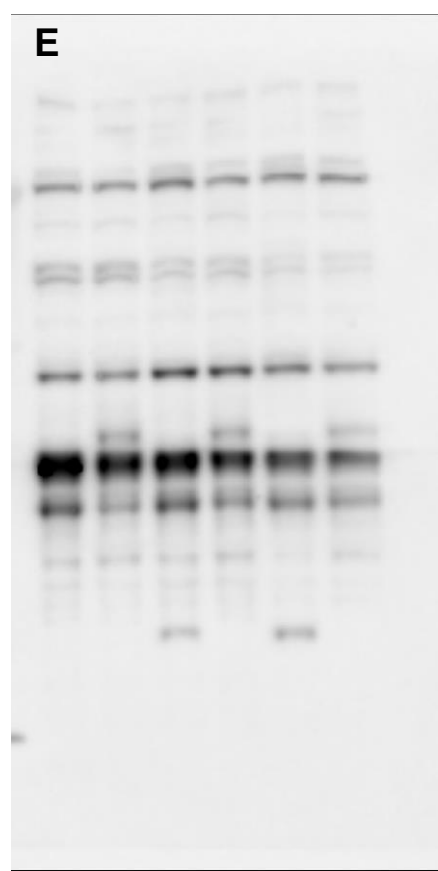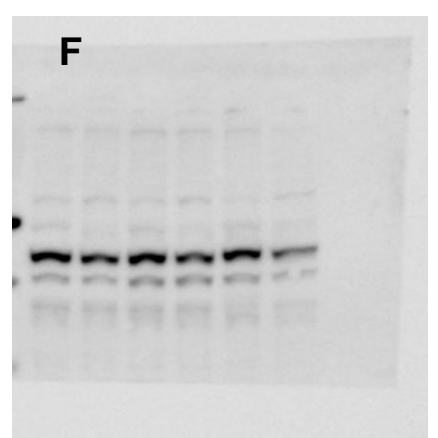

**G**

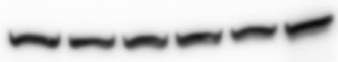

Supplement: Figure 3—source data 1. [file elife-84710-fig3-data1.pdf]

Figure 4—source data 1

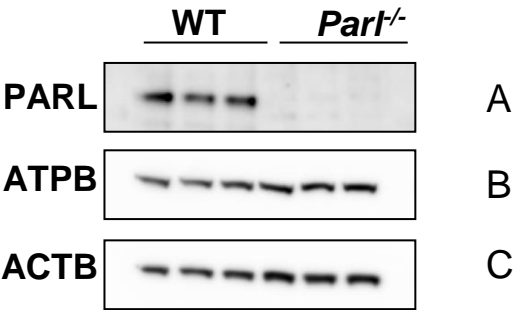

A

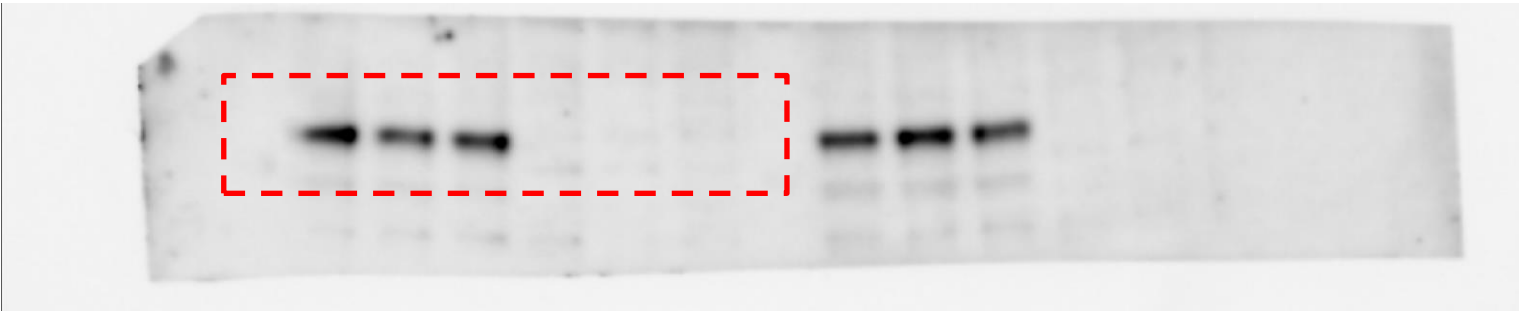

B

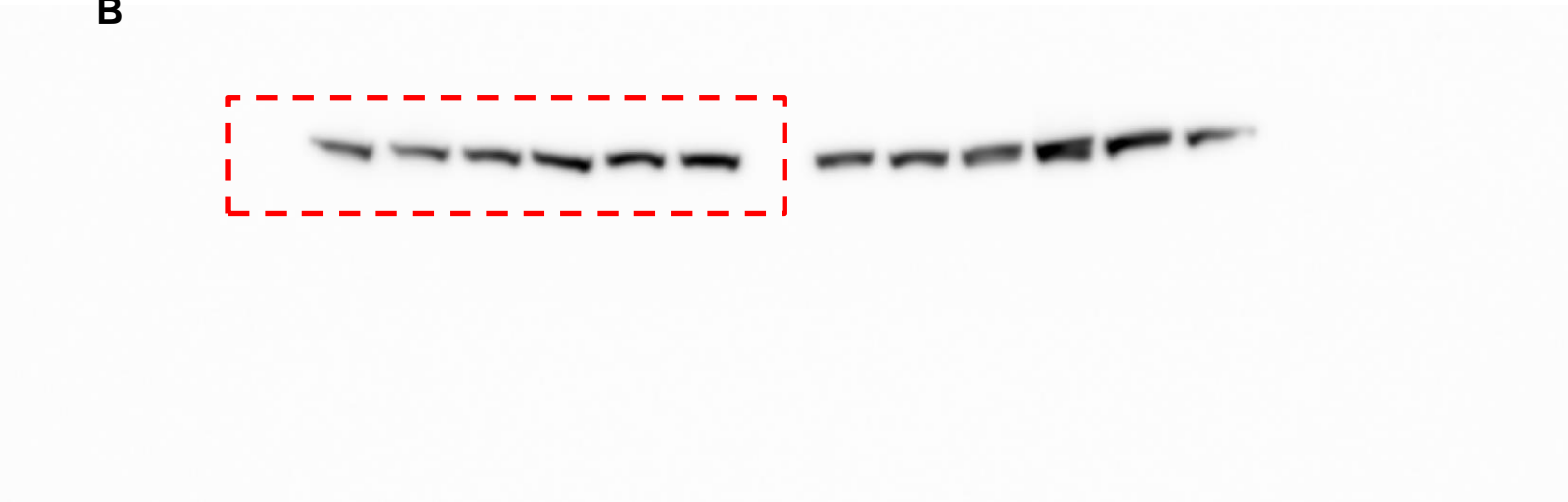

C

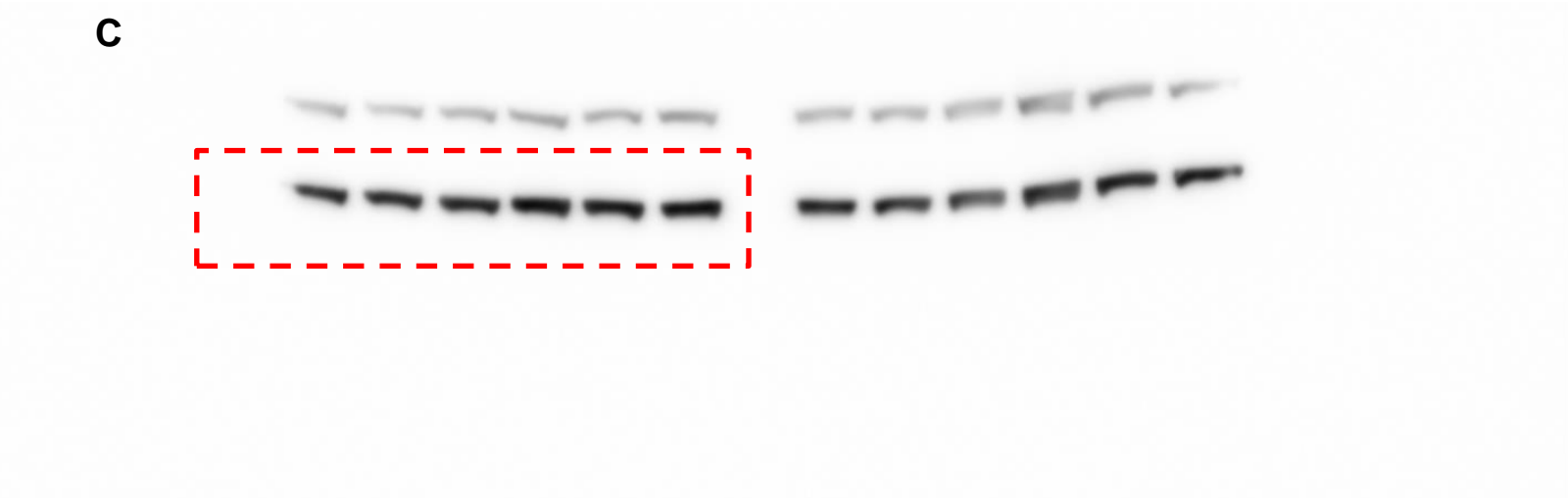

**A**

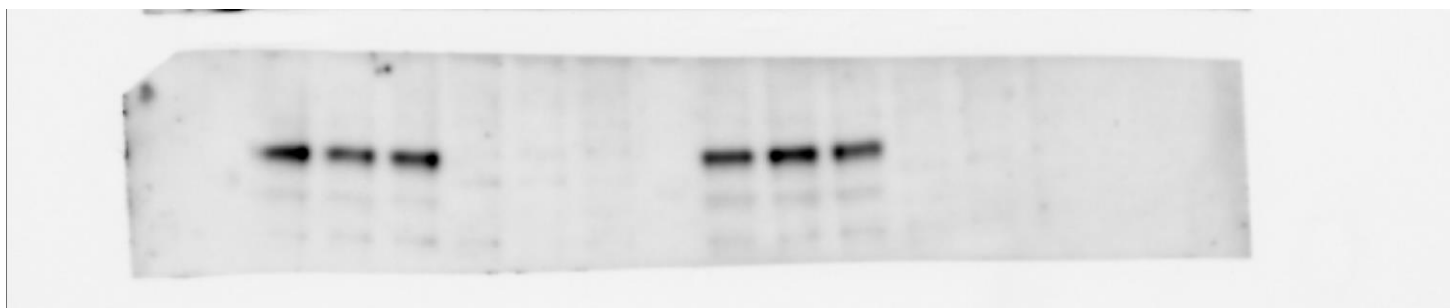

**B**

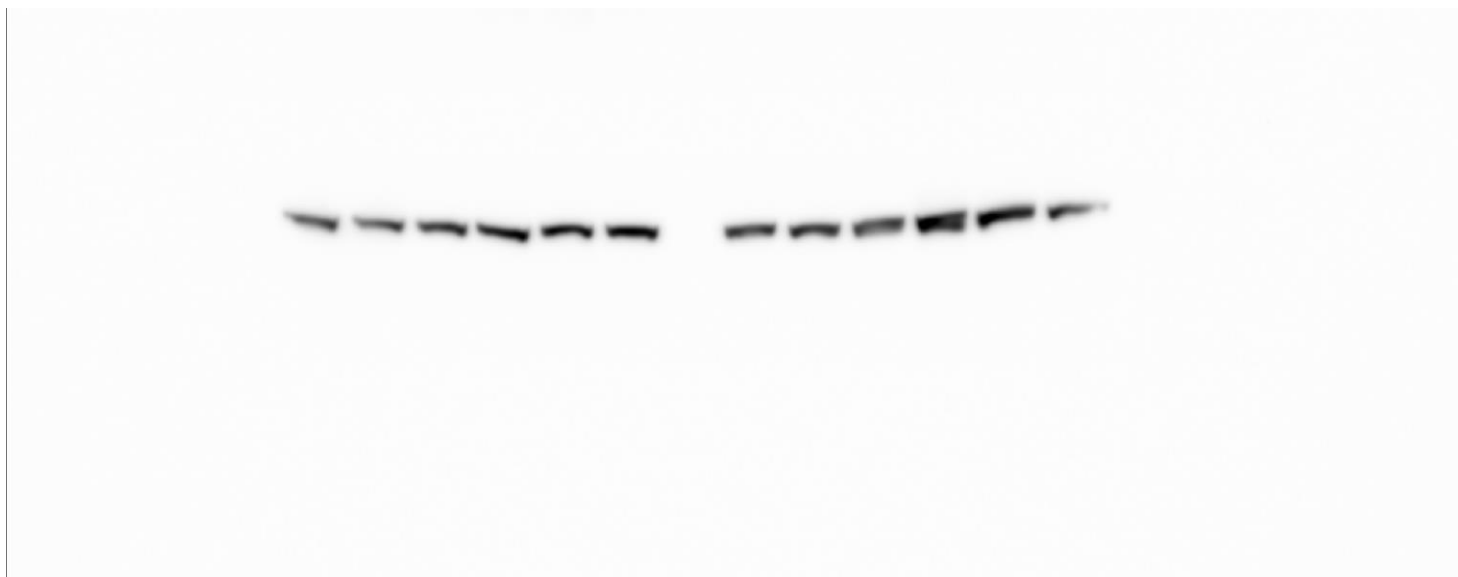

**C**

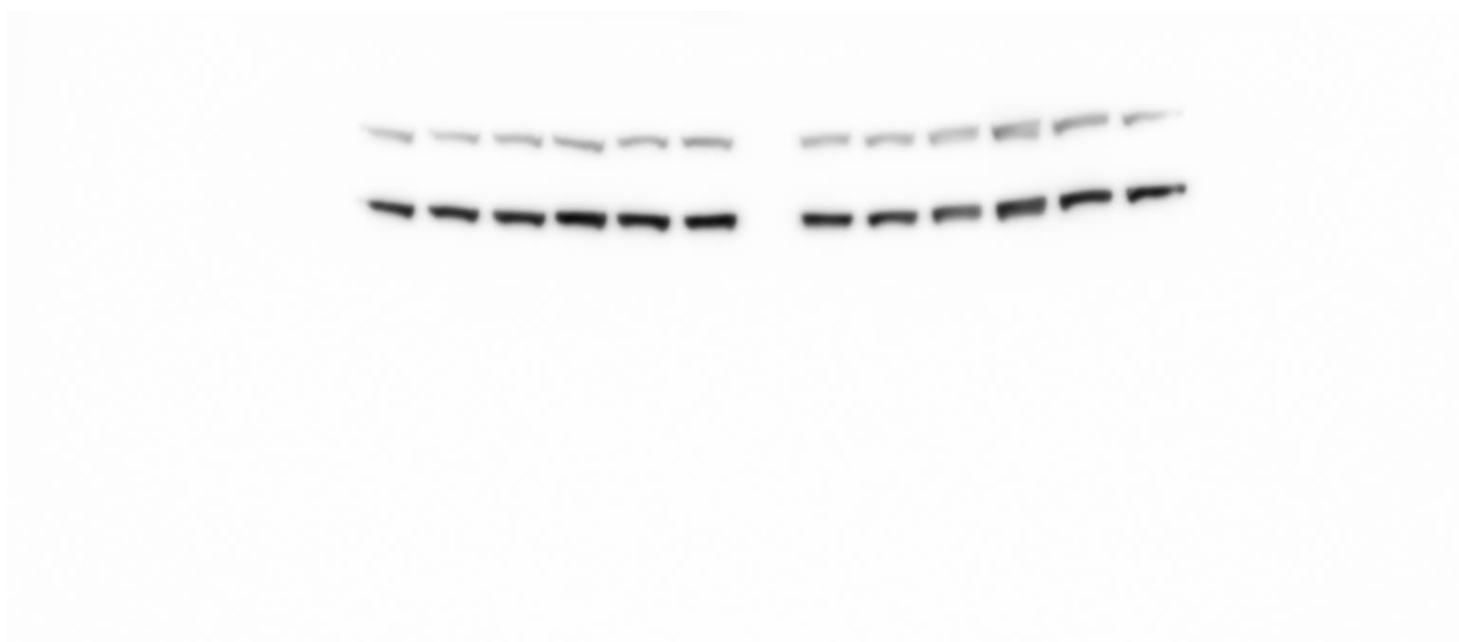

Supplement: Figure 4—source data 1. [file elife-84710-fig4-data1.pdf]

Figure 6—source data 1

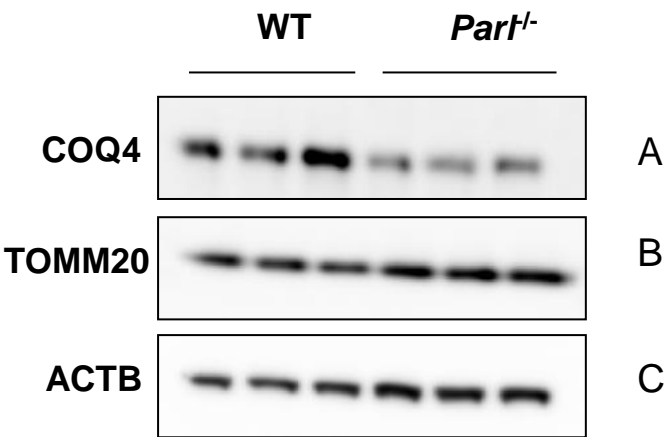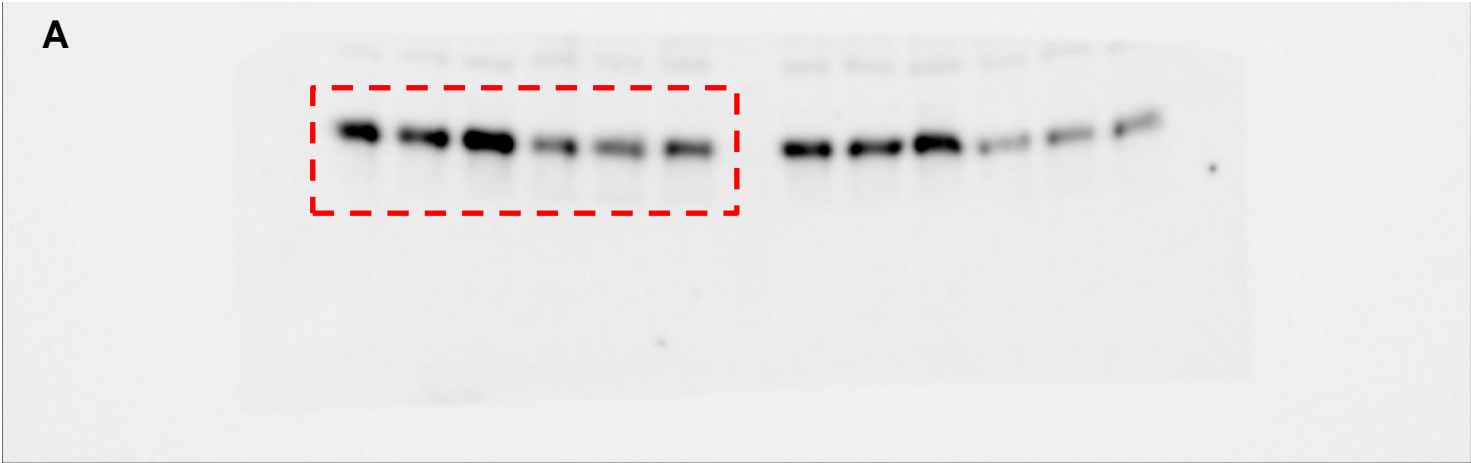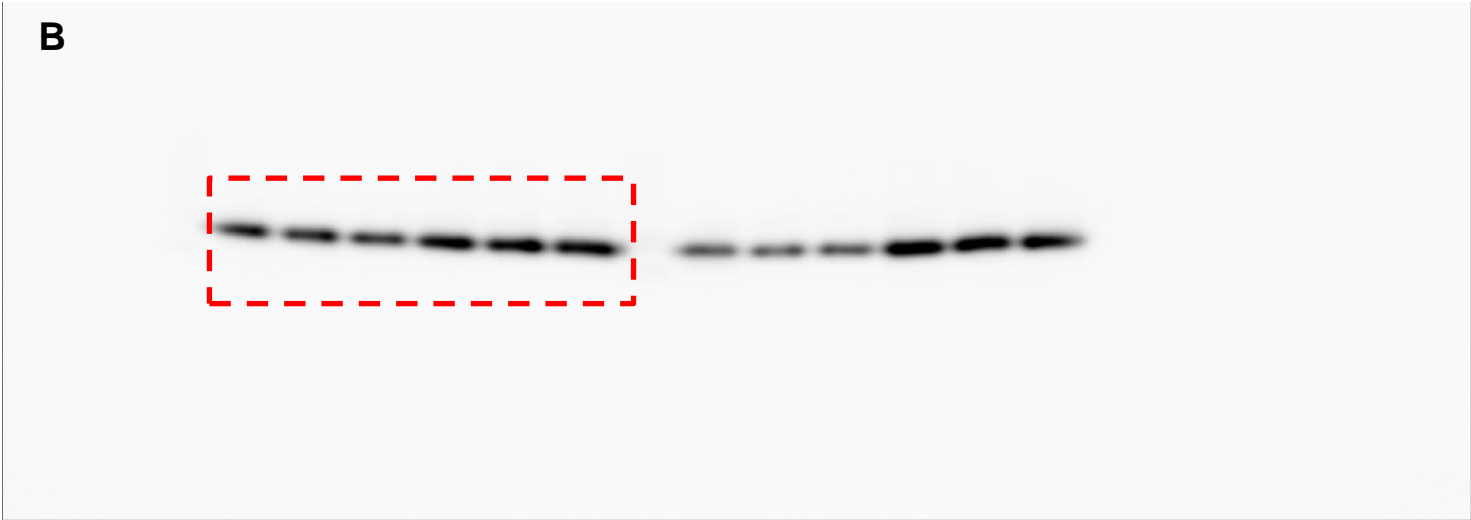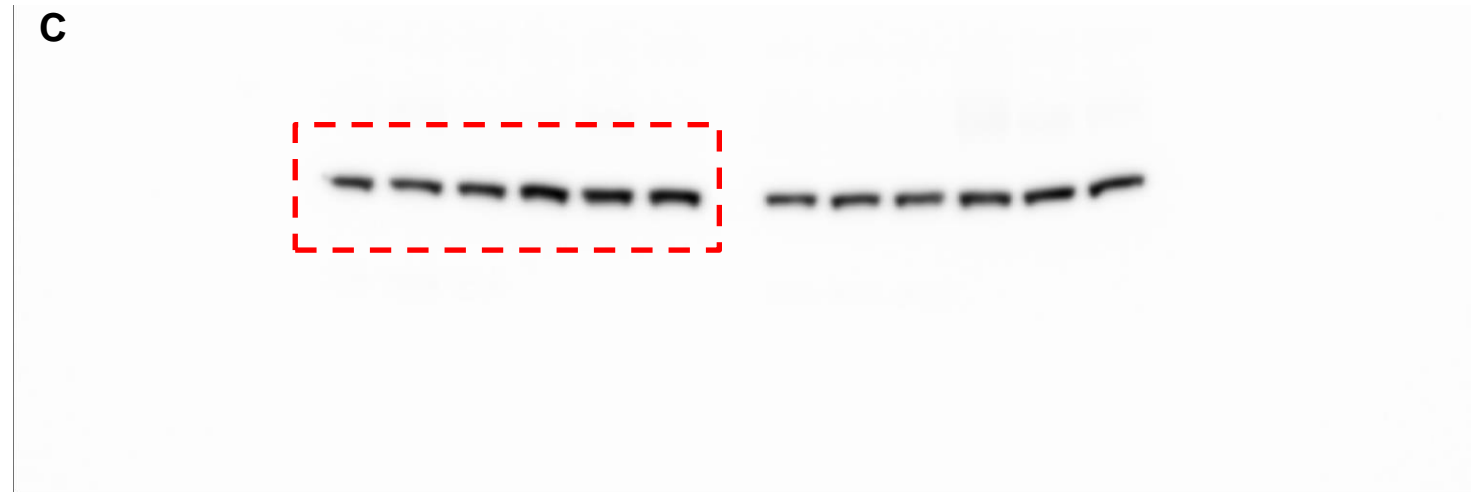

**A**

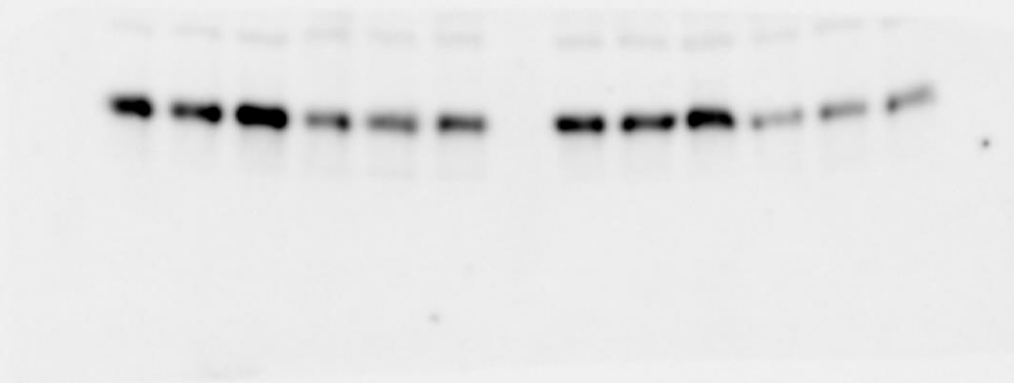

**B**

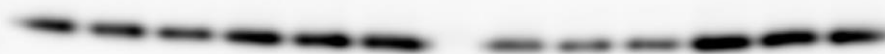

**C**

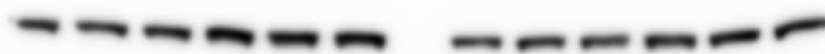

Supplement: Figure 6—source data 1. [file elife-84710-fig6-data1.pdf]

Figure 7—source data 1

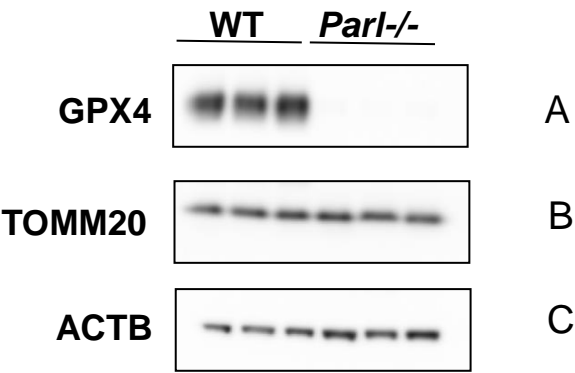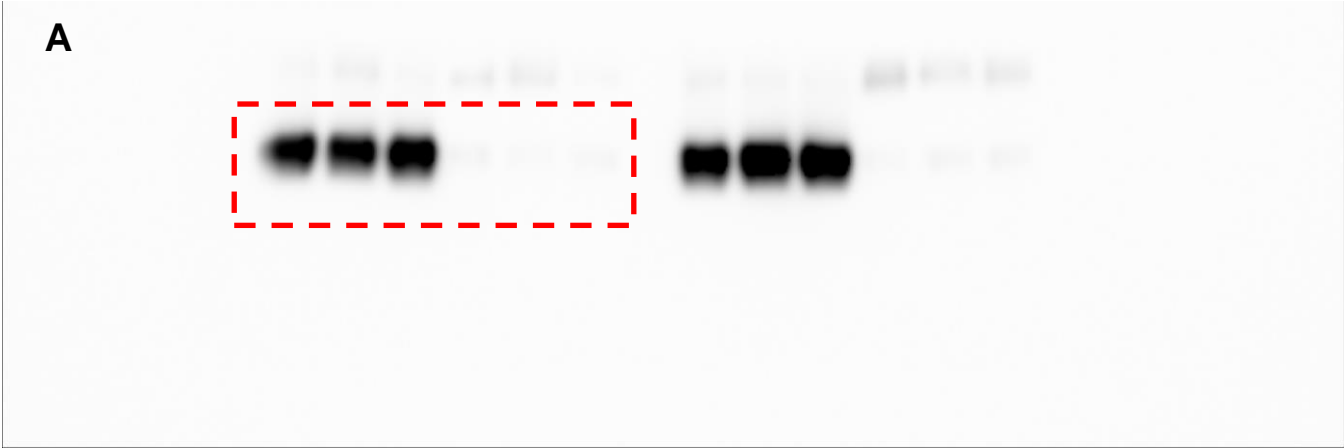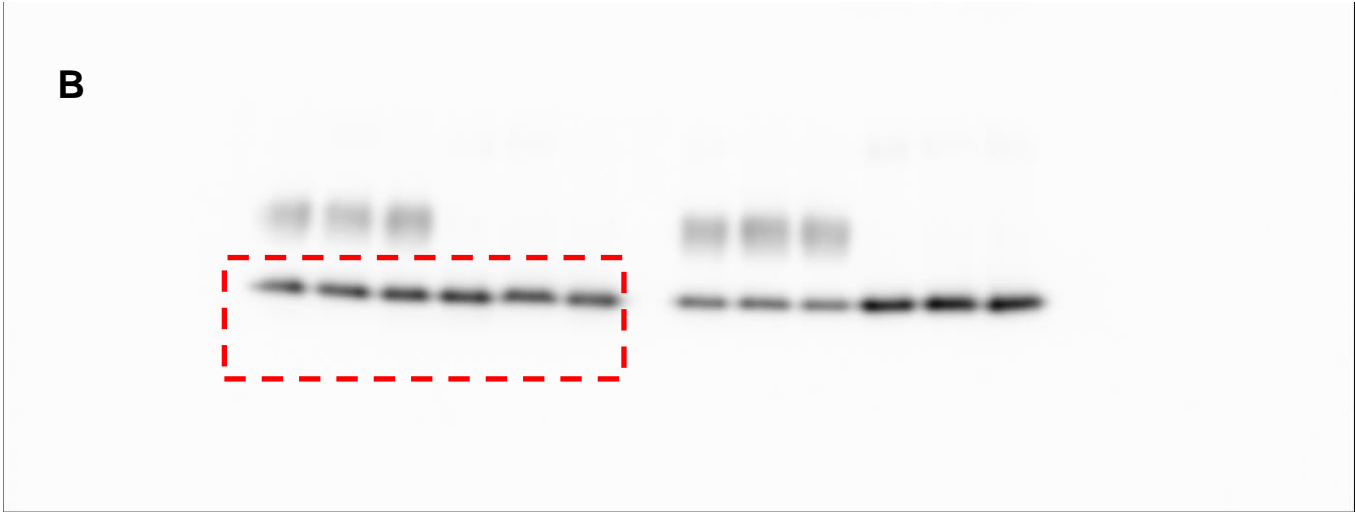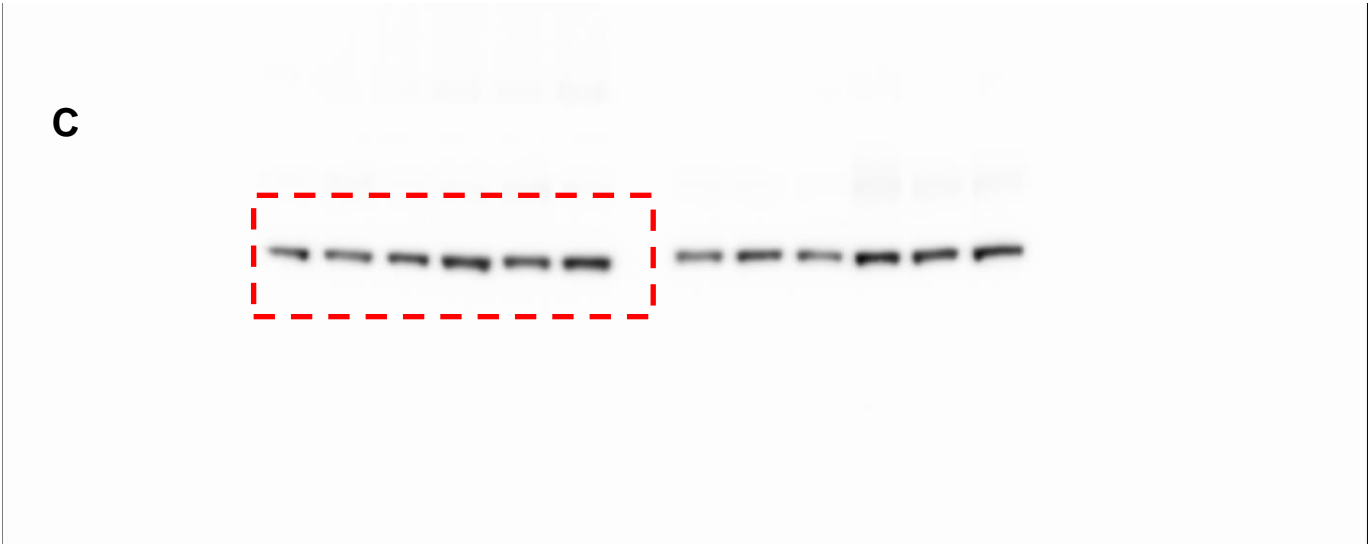

**A**

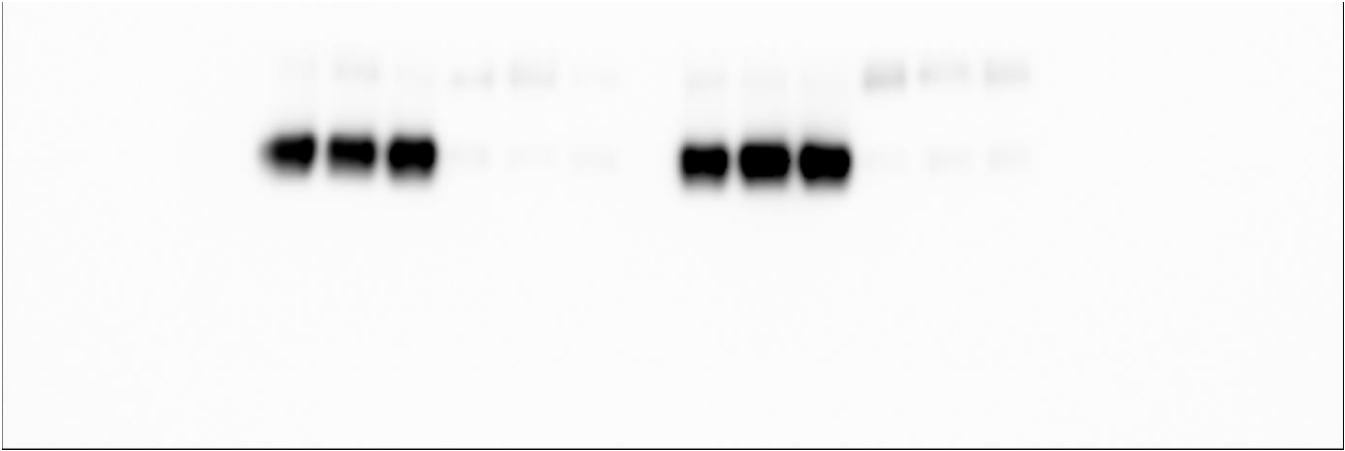

**B**

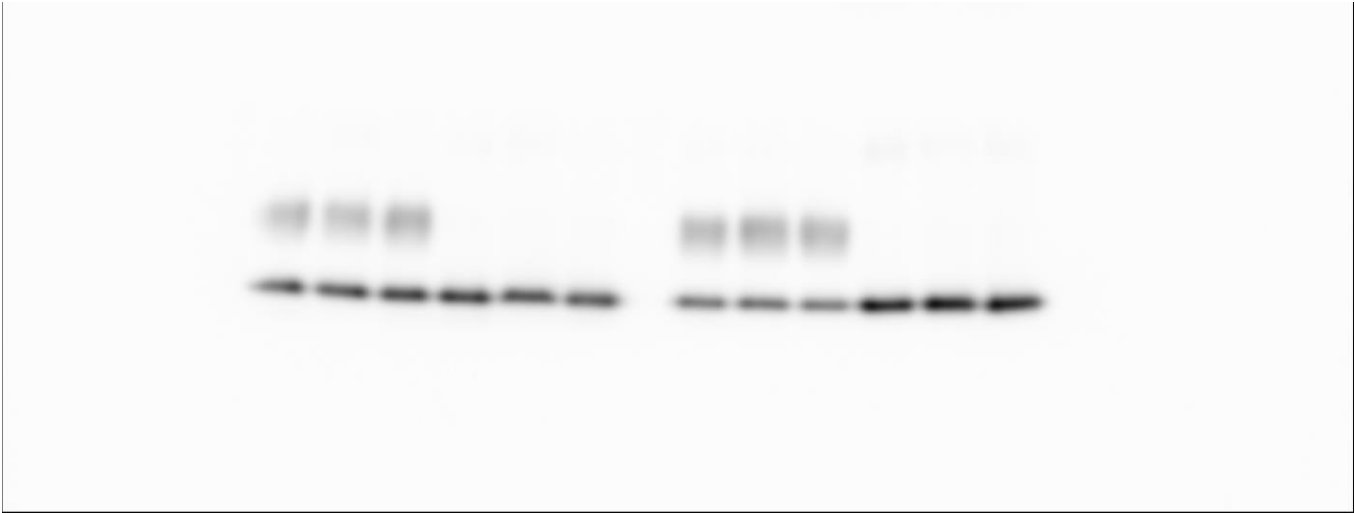

**C**

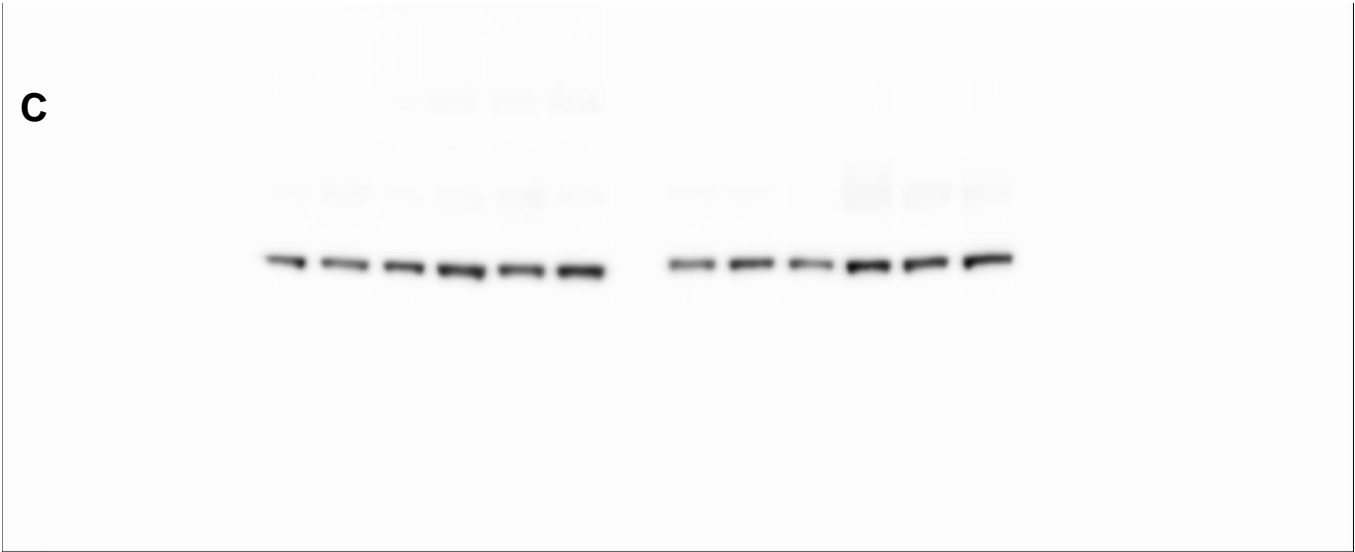

Supplement: Figure 7—source data 1. [file elife-84710-fig7-data1.pdf]

Figure 7—source data 2

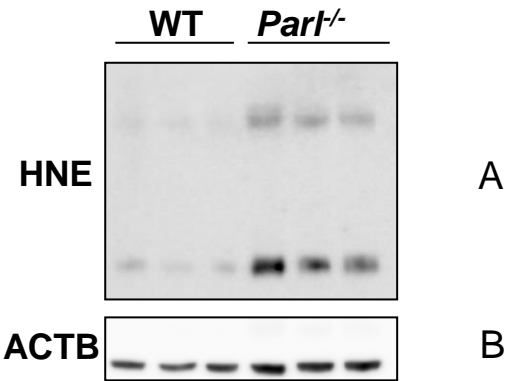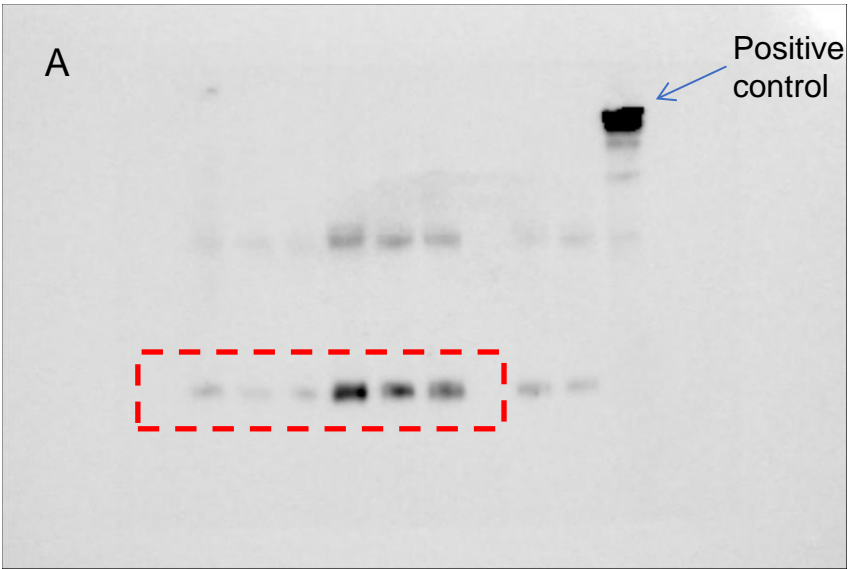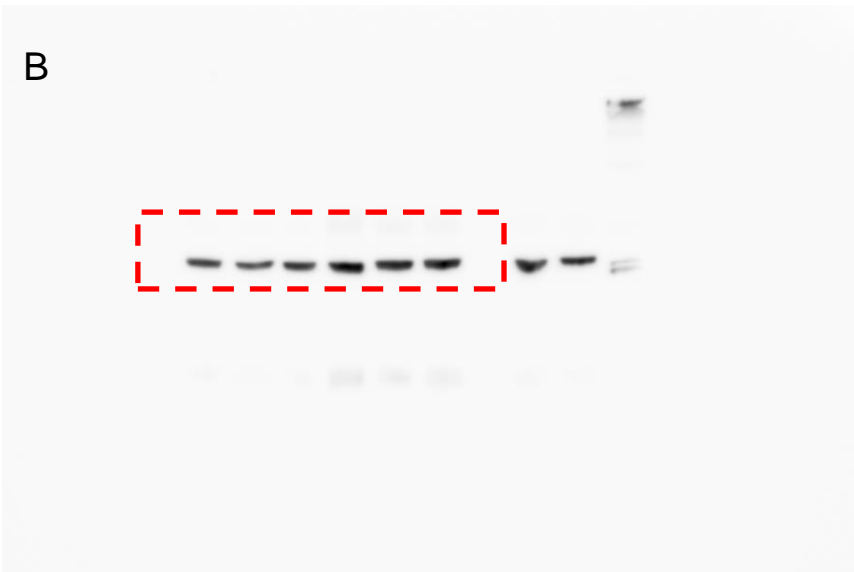

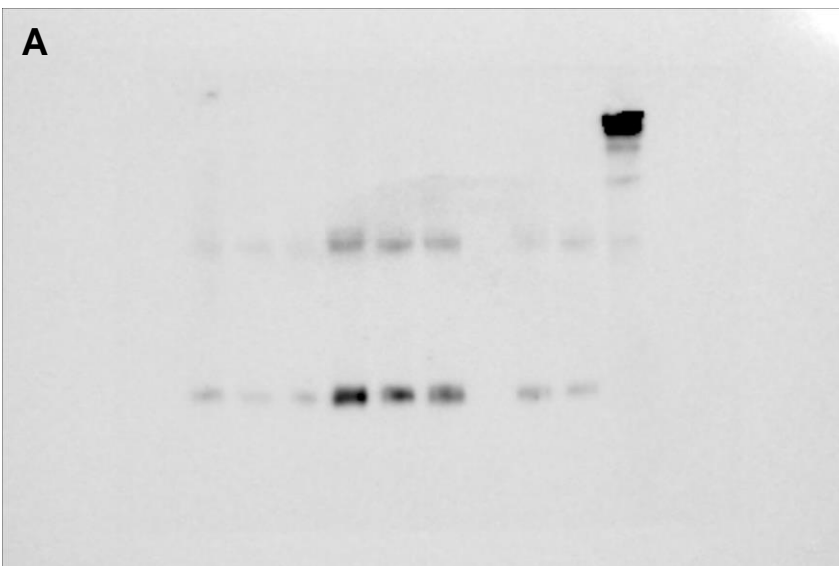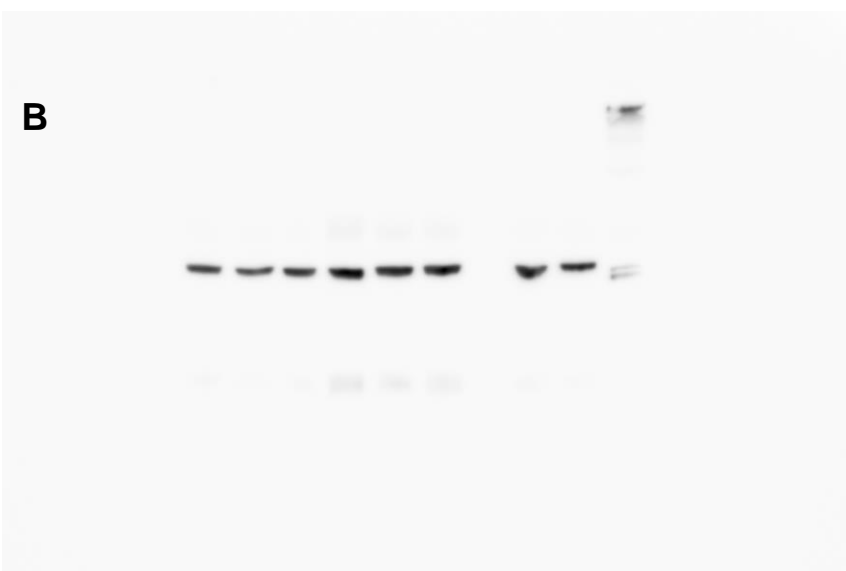

Supplement: Figure 7—source data 2. [file elife-84710-fig7-data2.pdf]

Figure 7-figure supplement 2-source data 1

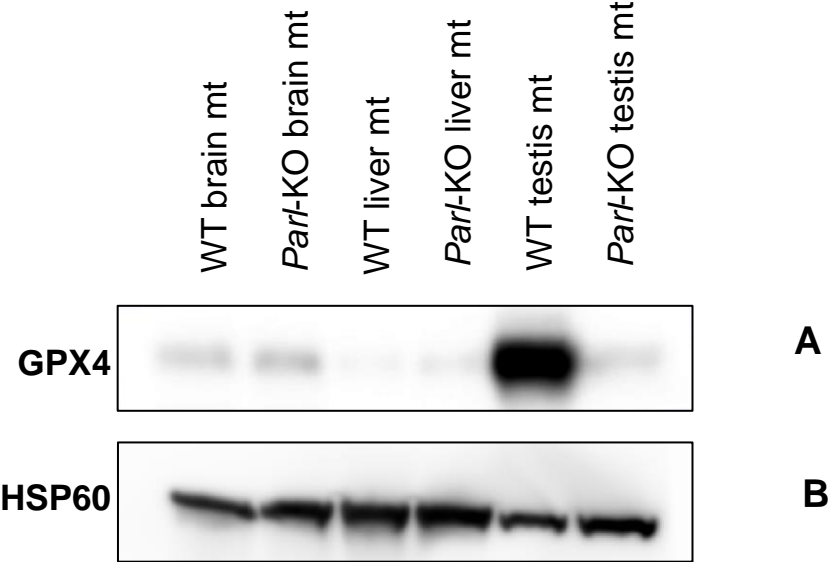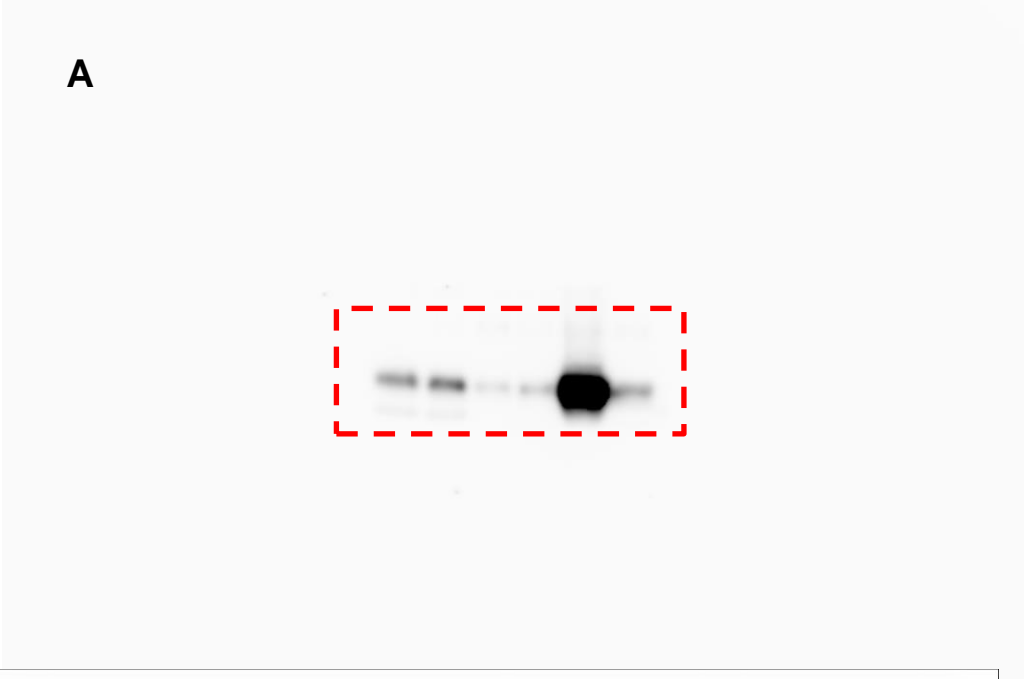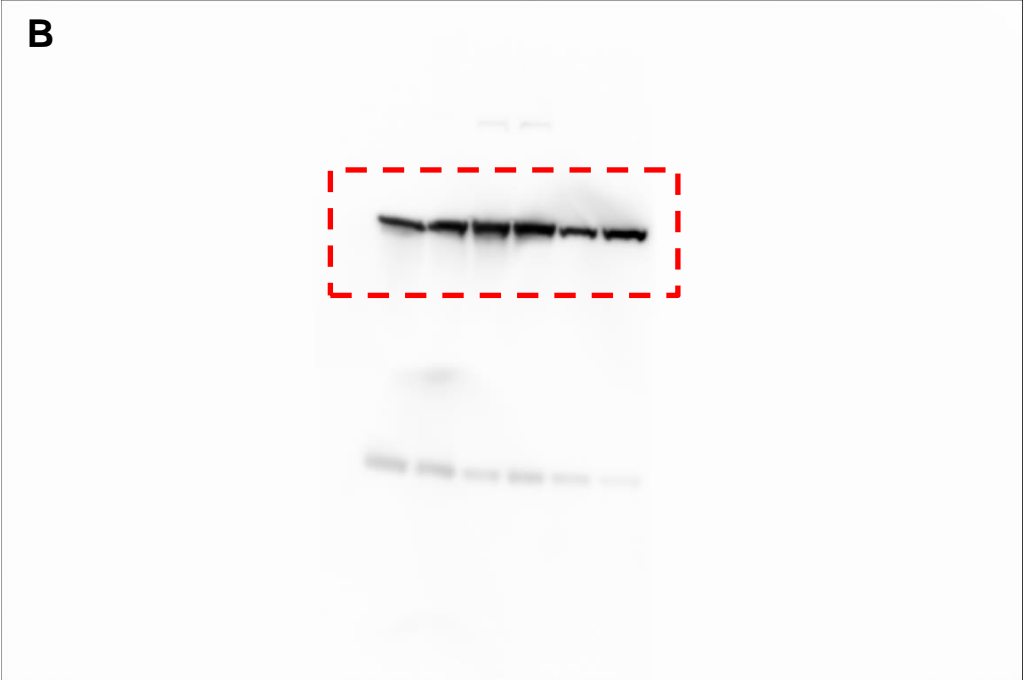

**A**

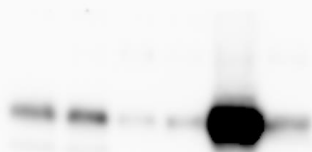

**B**

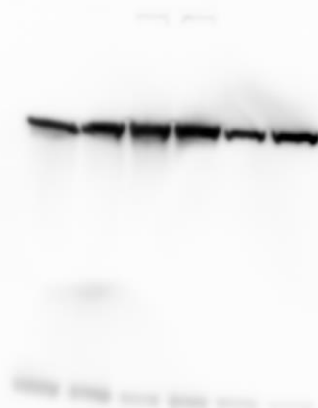

Supplement: Figure 7—figure supplement 2—source data 1. [file elife-84710-fig7-figsupp2-data1.pdf]

Figure 7-figure supplement 2-source data 2

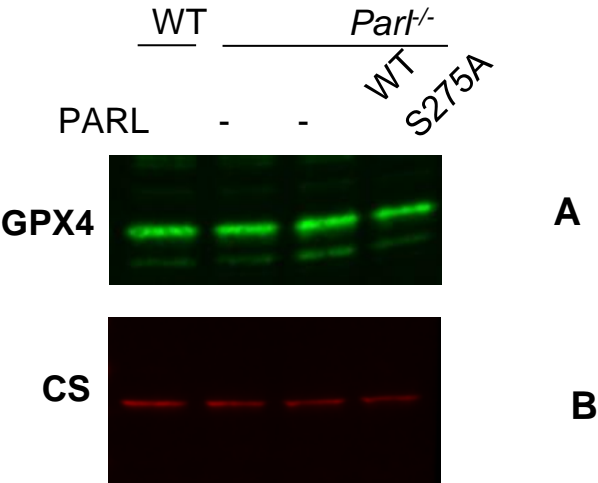

**A**

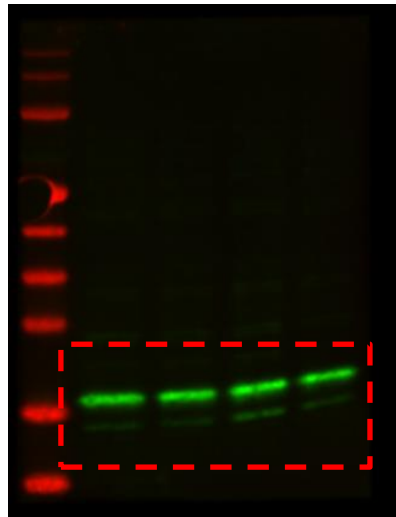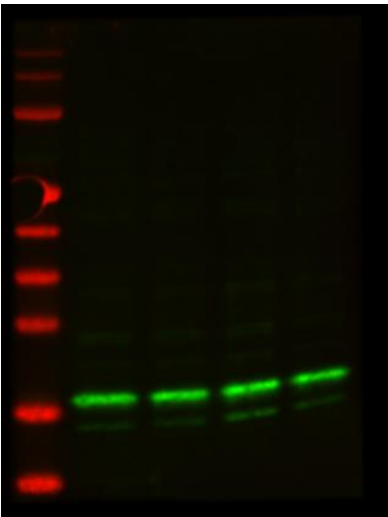

**B**

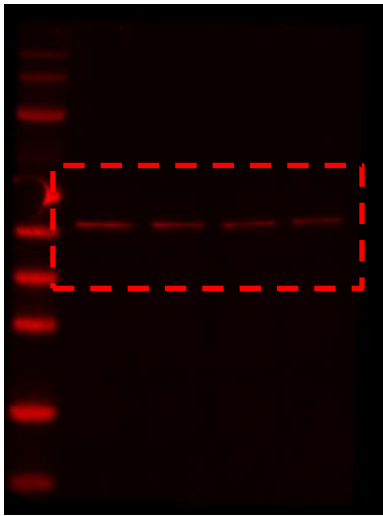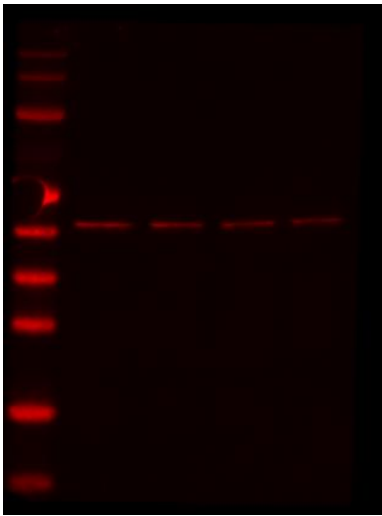

Supplement: Figure 7—figure supplement 2—source data 2. [file elife-84710-fig7-figsupp2-data2.pdf]

Figure 7-figure supplement 2-source data 3

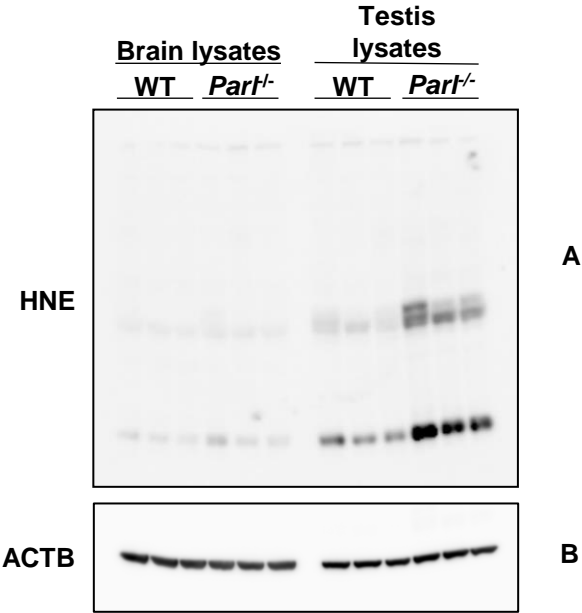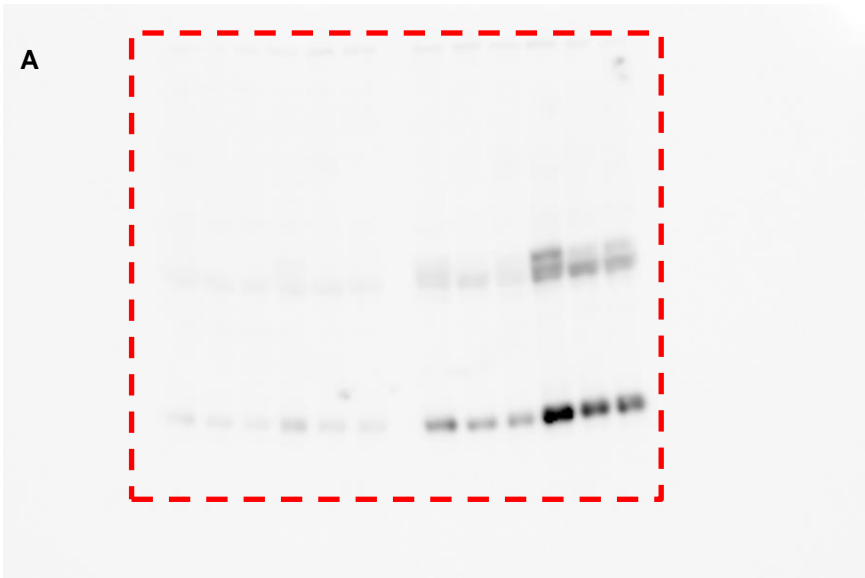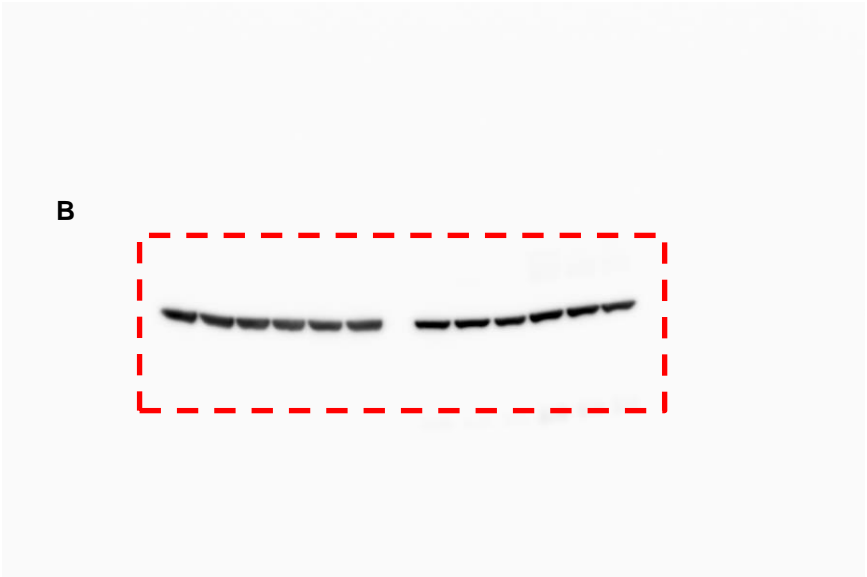

**A**

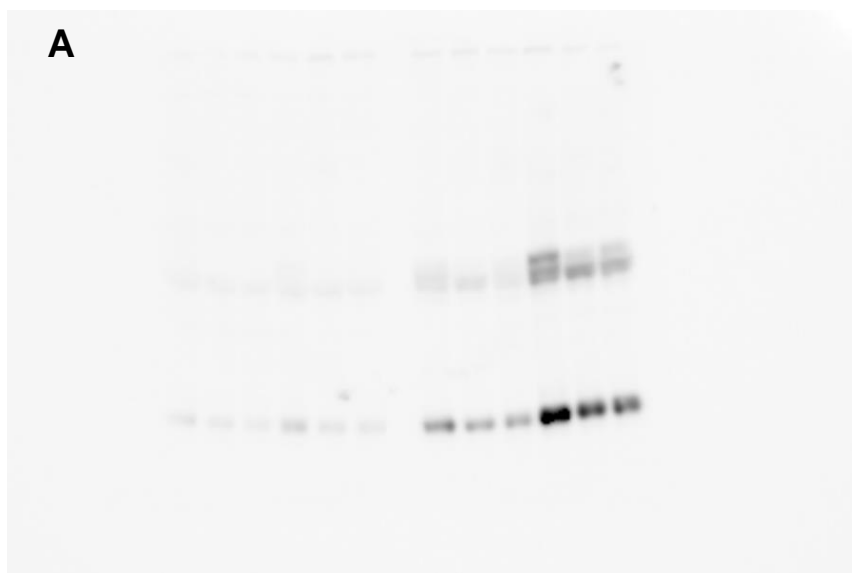

**B**

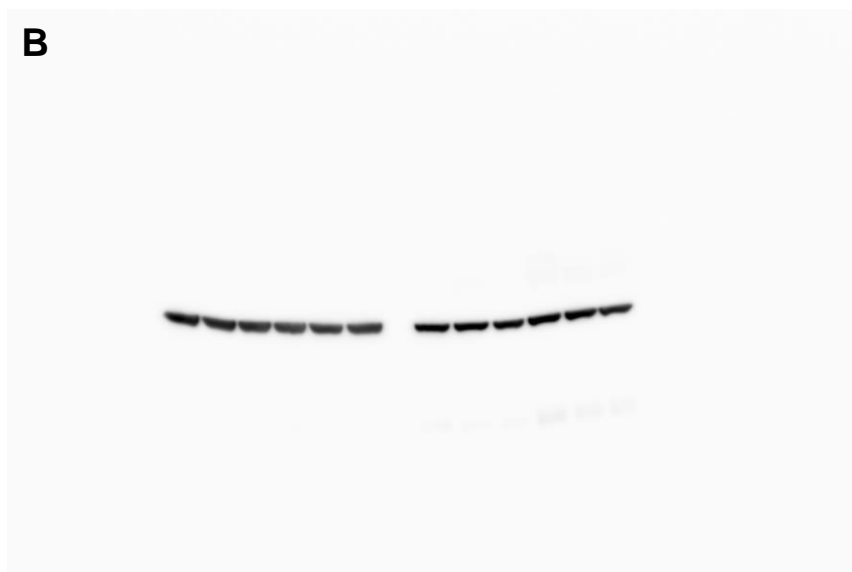

Supplement: Figure 7—figure supplement 2—source data 3. [file elife-84710-fig7-figsupp2-data3.pdf]

Figure 7-figure supplement 3-source data 1

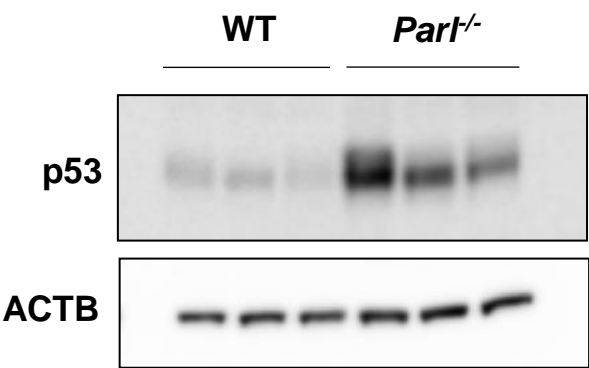

A

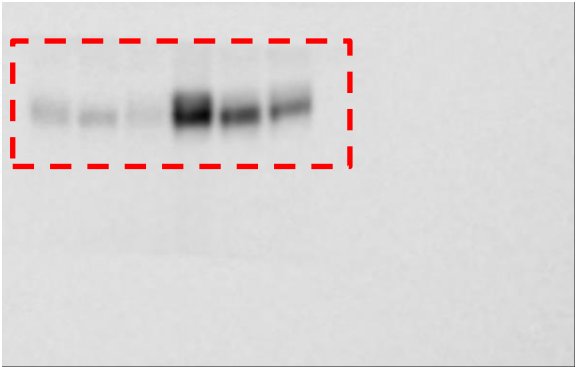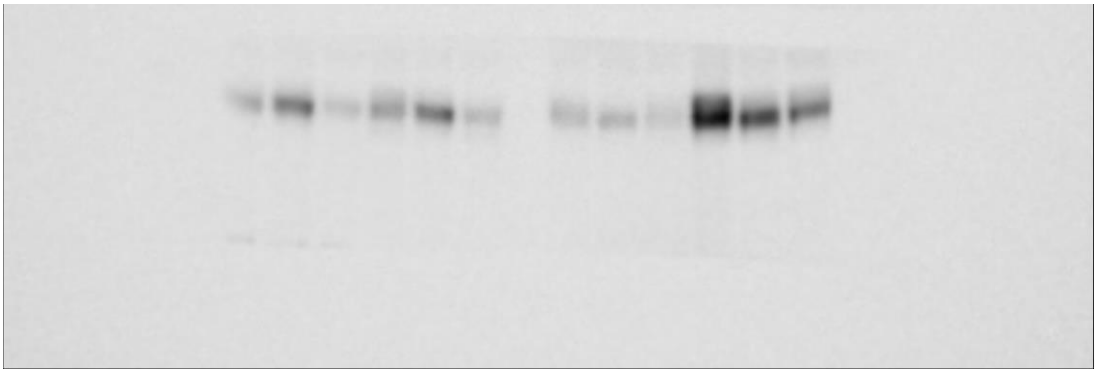

B

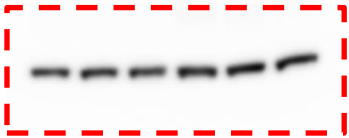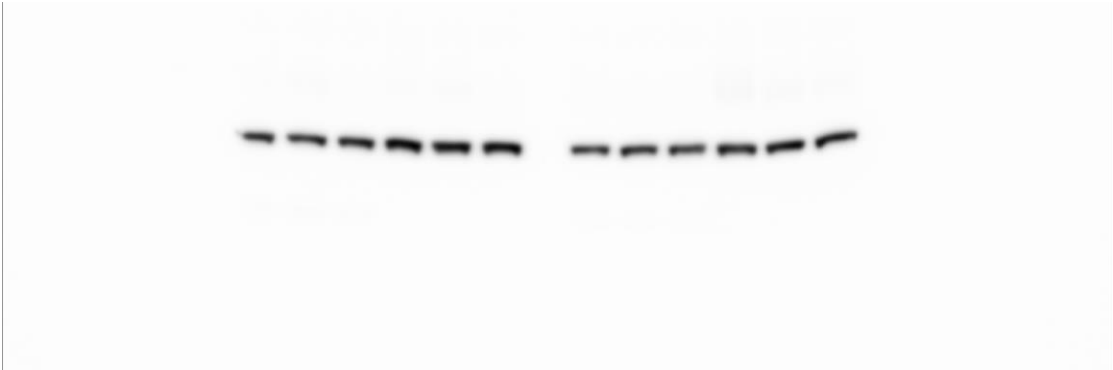

Supplement: Figure 7—figure supplement 3—source data 1. [file elife-84710-fig7-figsupp3-data1.pdf]
